# Population pharmacokinetics of ritonavir as a booster of lopinavir, atazanavir, or darunavir in African children with HIV

**DOI:** 10.1128/aac.00771-25

**Published:** 2025-09-26

**Authors:** Lufina Tsirizani, Hylke Waalewijn, Alexander Szubert, Veronica Mulenga, Chishala Chabala, Mutsa Bwakura-Dangarembizi, Moses Chitsamatanga, Diana A. Rutebarika, Victor Musiime, Mariam Kasozi, Abbas Lugemwa, Helen M. McIlleron, David M. Burger, Diana M. Gibb, Angela Colbers, Paolo Denti, Roeland E. Wasmann, the CHAPAS-4 trial team, Sarah Walker

**Affiliations:** 1 Division of Clinical Pharmacology, Department of Medicine, University of Cape Town, Cape Town, South Africa; 2 Training and Research Unit of Excellence, Kamuzu University of Health Sciences 37610, Blantyre, Malawi; 3 Medical Research Council Clinical Trials Unit at University College London 4919, London, United Kingdom; 4 Department of Paediatrics and Child Health, University of Zambia, School of Medicine 108234, Lusaka, Zambia; 5 University Teaching Hospital 217901 , Lusaka, Zambia; 6 University of Zimbabwe Clinical Research Centre 296969 , Harare, Zimbabwe; 7 University of Zimbabwe Faculty of Medicine and Health Sciences, Department of Child, Adolescent and Women’s Health 108329, Harare, Zimbabwe; 8 Department of Paediatrics, joint Clinical Research Centre, Kampala, Uganda; 9 Department of Paediatrics and Child Health, Makerere University, College of Health Sciences, School of Medicine 58588, Kampala, Uganda; 10 Department of HIV Research, joint Clinical Research Centre, Mbarara, Uganda; 11 Wellcome Centre for Infectious Diseases Research in Africa (CIDRI-Africa), Institute of Infectious Disease and Molecular Medicine, University of Cape Town, Cape Town, South Africa; 12 Department of Pharmacy, Radboudumc Research Institute for Medical Innovation (RIMI), Radboud University Medical Center, Nijmegen, the Netherlands; University Children's Hospital Münster, Münster, Germany

**Keywords:** population pharmacokinetics, ritonavir, NONMEM, pediatric antiretroviral, protease inhibitors

## Abstract

Ritonavir is important in antiretroviral therapy (ART) because it is used to boost the drug exposure of its fellow protease inhibitors (PIs). While PIs are commonly used in children, ritonavir data in this population are quite scarce. We investigated the population pharmacokinetics of ritonavir given to boost exposures of lopinavir, atazanavir, or darunavir, and co-administered with nucleoside reverse transcriptase inhibitors (NRTIs) in African children, and investigated factors affecting its exposure. We conducted a pharmacokinetic sub-study within the CHAPAS-4 (ISRCTN22964075) trial, which randomized children to two NRTIs with twice-daily lopinavir/ritonavir, once-daily atazanavir/ritonavir, or once-daily darunavir/ritonavir, as second-line ART. Intensive pharmacokinetic blood samples were collected at week 6, and nonlinear mixed-effects modeling was used to identify factors affecting ritonavir pharmacokinetics. In all, 170 children were enrolled in the ritonavir-boosted PI arms of the CHAPAS-4 pharmacokinetic sub-study, with median age 10.6 (range 3.2–15.6) years and weight 26.0 (14.2–64.2) kg. Despite similar dose levels, ritonavir exposure varied widely depending on the companion PI. Compared to children on darunavir/ritonavir, those on atazanavir/ritonavir had 137% (95% CI 107%–190%) higher bioavailability and 20% (95% CI 11.3%–31.3%) faster clearance, while those on lopinavir/ritonavir had 23.4% (95% CI 8.20%–34.4%) lower bioavailability. No effect of NRTIs on ritonavir pharmacokinetics was observed. Ritonavir exposure is higher with atazanavir than with lopinavir or darunavir. These data provide greater insight into the use of ritonavir for boosting PIs in children and help reduce the knowledge gap regarding its exposure in children.

## INTRODUCTION

Ritonavir is a protease inhibitor (PI) with activity against HIV (1), but due to its neurological and gastrointestinal side effects at therapeutic doses, it is only used at low doses to boost the exposures of other drugs like integrase strand transfer inhibitors (INSTIs) and other PIs, by inhibiting their metabolism (2). Low-dose ritonavir strongly inhibits gut and liver CYP3A4 as well as the activity of P-glycoprotein efflux transporter (3), meaning that, when combined with other PIs, they can be given at lower doses and less frequently than without ritonavir (1). Ritonavir is itself metabolized by CYP3A4 and CYP2D6 and induces its own metabolism. It is well absorbed after oral administration and is approximately 99% plasma protein bound to alpha-1-acid glycoprotein and albumin (4). Ritonavir-boosted PIs (bPI) play a significant role in first-, second-, and third-line pediatric antiretroviral therapy (ART) (5).

Currently, children newly diagnosed with HIV are started on first-line ART, which comprises either dolutegravir plus two nucleoside reverse transcriptase inhibitors (NRTIs) if aged >4 weeks and weighing >3 kg or ritonavir-boosted lopinavir with two NRTIs (6). The NRTIs of choice in children are abacavir (ABC) and lamivudine (3TC), or alternatively, zidovudine (ZDV) and lamivudine (3TC) (6). Children failing on first-line ART are started on second-line ART. If the child was on dolutegravir-based ART during first-line therapy, they are recommended to switch to a ritonavir-boosted PI (lopinavir, atazanavir, or darunavir) plus two optimized NRTI (6). If the child was not on dolutegravir-based ART, dolutegravir plus two optimized NRTIs is recommended as second-line therapy. Those failing on second-line therapy are typically started on ritonavir-boosted darunavir co-administered with dolutegravir and two optimized NRTIs, as third-line therapy (5, 7).

As can be noted, ritonavir-boosted PIs form a significant part of pediatric ART regimens. Despite their wide use in children, data describing the pharmacokinetics and exposure of ritonavir used to boost these PIs in children are scarce (5). When previously tested as a lone PI at higher doses (200–500 mg), ritonavir exhibited dose-dependent saturable pharmacokinetics (4). It is therefore important to understand its kinetics when administered at lower doses for boosting other PIs in children. Previous studies investigating the pharmacokinetics of ritonavir have primarily focused on adult populations (1, 4, 8, 9). It is widely recognized that drug exposures and drug-drug interactions observed in adults may not directly translate to children (10). Studies that looked at the pharmacokinetics of ritonavir-boosted PIs in children were generally small and did not compare the pharmacokinetics of ritonavir across different boosted PIs (11). For example, in adults, atazanavir has been shown to increase the exposure of other PIs and may therefore significantly affect the exposure of ritonavir (12). Additionally, different boosted PIs vary in their affinity for CYP3A4 and are known to be inducers and/or inhibitors of CYP450 enzymes and transporters, thus potentially affecting ritonavir exposure to varying degrees (13, 14). Understanding the pharmacokinetics of PI-boosting ritonavir and evaluating factors that affect its exposure would fill the gap in information and give more insight into its use in children. Furthermore, data on the concomitant use of ritonavir-boosted PIs with newer NRTIs like tenofovir alafenamide (TAF) are scarce (15).

In this study, we describe the population pharmacokinetics of ritonavir given to boost lopinavir, atazanavir, or darunavir in African children enrolled in the CHAPAS-4 study. We also explore the effect of the boosted PIs, TAF, standard of care NRTIs, age, and body size on the pharmacokinetics of ritonavir in children.

## MATERIALS AND METHODS

### Study design and participants

CHAPAS-4 (ISRCTN22964075) was an open-label, multicenter, 4 × 2 randomized trial evaluating the virological efficacy and pharmacokinetics of lopinavir/ritonavir, darunavir/ritonavir, atazanavir/ritonavir, and dolutegravir, in combination with either Tenofovir Alafenamide/emtricitabine (TAF/FTC) or standard of care (SOC) backbones containing abacavir/lamivudine (ABC/3TC) or zidovudine/lamivudine (ZDV/3TC) (16).

The trial included children with HIV from Zambia, Uganda, and Zimbabwe, aged 3–15 years, weighing at least 14 kg (Table 1), receiving abacavir-, zidovudine-, or stavudine-containing NRTI backbone and failing on ART according to WHO criteria (confirmed viral load >1,000 copies/mL after adherence counseling or CD4 criteria for failure or clinical criteria for failure). A nested pharmacokinetic sub-study was conducted within the CHAPAS-4 trial. Children with illness(es) that could affect pharmacokinetic results, and those using medication with known drug-drug interaction with the trial drugs, were not eligible for inclusion. Written informed consent was given by parents/caregivers, and local and national ethics committees approved the main trial and nested pharmacokinetic sub-study.

**TABLE 1:** Baseline characteristics of children who received ritonavir in the CHAPAS-4 study, stratified by boosted protease inhibitor arm^*a*^

	Lopinavir/ritonavir (*N* = 51)	Atazanavir/ritonavir (*N* = 60)	Darunavir/ritonavir (*N* = 59)	Total (*N* = 170)
Age (years)				
Median [Min–Max]	10.3 [4.27–14.7]	9.85 [3.16–15.6]	10.9 [3.83–14.7]	10.5 [3.16–15.6]
Sex				
Female	26 (51.0%)	28 (46.7%)	33 (55.9%)	87 (51.2%)
Male	25 (49.0%)	32 (53.3%)	26 (44.1%)	83 (48.8%)
Weight (kg)				
Median [Min–Max]	26.0 [14.2–49.5]	26.6 [14.5–64.2]	26.0 [14.5–47.0]	26.0 [14.2–64.2]
Weight band				
14–19.9 kg	11 (21.6%)	12 (20.0%)	12 (20.3%)	35 (20.6%)
20–24.9 kg	12 (23.5%)	16 (26.7%)	17 (28.8%)	45 (26.5%)
25–34.9 kg	13 (25.5%)	18 (30.0%)	17 (28.8%)	48 (28.2%)
35 + kg	15 (29.4%)	14 (23.3%)	13 (22.0%)	42 (24.7%)
Height (cm)				
Median [Min–Max]	132 [97.0–169]	129 [102–162]	132 [97.8–161]	131 [97.0–169]
Weight for age Z-scores				
Median [Min–Max]	−1.2 [−4.4 to 0.5]	−1.1 [−4.1 to 1.7]	−1.7 [−4.5 to 1.0]	−1.4 [−4.5 to 1.7]
Height for age Z-scores				
Median [Min–Max]	−1.2 [−4.4 to 1.2]	−1.3 [−4.0 to 3.6]	−1.5 [−4.3 to 2.1]	−1.3 [−4.4 to 3.6]
RTV total daily dose (mg)				
Median [Min–Max]	100 [50.0–100]	100 [75.0–100]	100 [100–100]	100 [50.0–100]
RTV total daily dose (mg/kg)				
Median [Min–Max]	2.65 [2.02–4.00]	3.41 [1.56–6.90]	3.96 [2.13–6.90]	3.25 [1.56–6.90]
RTV Formulation				
100 mg RTV only	0 (0%)	4 (6.7%)	59 (100%)	63 (37.1%)
75 mg RTV only	0 (0%)	24 (40.0%)	0 (0%)	24 (14.1%)
100 mg co-formulated with 300 mg ATV	0 (0%)	32 (53.3%)	0 (0%)	32 (18.8%)
50 mg co-formulated with 200 mg LPV	51 (100%)	0 (0%)	0 (0%)	51 (30.0%)
NRTI backbone				
TAF/FTC	25 (49.0%)	34 (56.7%)	34 (57.6%)	93 (54.7%)
ABC/3TC	14 (27.5%)	17 (28.3%)	12 (20.4%)	43 (25.3%)
ZDV/3TC	12 (23.5%)	9 (15.0%)	13 (22.0%)	34 (20.0%)

^*a*^ TAF/FTC, Tenofovir alafenamide/emtricitabine; ABC/3TC, Abacavir/lamivudine, ZDV/3TC; Zidovudine/lamivudine, RTV, ritonavir; ATV, atazanavir; LPV, lopinavir.

### Procedures

Ritonavir was given in tablet form, alone or co-formulated with companion PIs and dosed according to the following weight bands: 14–19.9, 20–24.9, 25–34.9, and ≥35 kg. The dose depended on the companion PI (Table 2). NRTI backbones were also dosed according to weight bands. Children were followed up for 96 weeks. Pharmacokinetic blood samples were collected after week 6 of study treatment. Samples were collected at pre-dose, 0.5 (only for those on TAF/FTC), 1, 2, 4, 6, 8, 12, and 24 hours post-dose, and plasma was stored at −80°C. On the day of pharmacokinetic blood sampling, study drugs were administered under direct observation, with food (breakfast with 5% fat, ~250 kCal). All non-ART co-medications were taken at least 2 hours after study drugs. Ritonavir plasma concentrations were measured at the Department of Pharmacy, Radboud University Medical Center, Nijmegen, the Netherlands using a validated high-performance liquid chromatography quantification method with a lower limit of quantification (LLOQ) of 0.045 mg/L. All the original values of detectable drug concentrations (even if below the limit of quantification) were reported by the laboratory and available for the analysis.

**TABLE 2:** Ritonavir dosing and drug formulations used in the CHAPAS-4 study^*b*^

WHO weight bands	Lopinavir (LPV)/ritonavir (r)		Atazanavir (ATV)/ritonavir (r)				Darunavir (DRV)/ritonavir (r)			
	200/50 mg LPV/r	Daily dose (mg) LPV/r	100 mg ATV	25 mg R	300/100 mg ATV/r	Daily dose (mg) ATV/r (AM)	150 mg DRV	400 mg DRV	100 mg r	Daily dose (mg) DRV/r (AM)
	Number of tablets BD (AM + PM)		Number of tablets OD	Number of tablets OD	Number of tablets OD		Number of tablets OD	Number of tablets OD	Number of tablets OD	
14–19.9 kg	1 + 1	400/100	2	3^*a*^	–^*c*^	200/75	4	–	1	600/100
20–24.9 kg	1 + 1	400/100	2	3^*a*^	–	200/75	4	–	1	600/100
25–34.9 kg	2 + 1	600/150	–	–	1	300/100	–	2	1	800/100
≥35 kg	2 + 2	800/200	–	–	1	300/100	–	2	1	800/100

^*a*^ If 25 mg ritonavir was not available, 100 mg ritonavir was used to boost atazanavir for the 14–19.9 kg and 20–24.9 kg weight bands.

^*b*^ OD, once daily; BD, twice daily; AM, morning; PM, evening.

^*c*^ –, not applicable.

### Population pharmacokinetic analysis

Population pharmacokinetic analysis was used to analyze concentration-time data of ritonavir using the first-order conditional estimation with interaction (FOCE-I) algorithm in NONMEM v7.5.1 (ICON Development Solutions, Ellicott City, MD, USA). R v4.2.2 via RStudio (Posit Public Benefit Corporation) and Pirana v2.9.9 (Certara, Inc.) were utilized to visualize and process the data, and Perl Speaks NONMEM v5.2.6 (Department of Pharmacy, Uppsala University) was used for automation of the analysis processes (17).

We tested one- and two-compartment disposition models and several absorption models (sequential zero- and/or first-order, with lag time or transit compartments [18]). Simple first-order elimination as well as first-pass and saturable elimination through a liver compartment were tested. We included between-subject variability (BSV) on disposition parameters and between-occasion variability (BOV) on absorption parameters, assuming a log-normal distribution. Combined additive and proportional error models to describe residual unexplained variability were evaluated. Samples with undetectable drug concentrations were treated as censored at the limit of detection (LOD), which was imputed as 30% of the LLOQ (19), and handled using a modified version of the M6 method by Beal (20). The undetectable (i.e., censored) values were imputed to half of the LOD value (LOD/2), and the additive error was inflated by LOD/2. If a series of undetectable values was present, the value closest to the peak concentration was retained, while all trailing values were excluded from the model fit but retained for simulation-based diagnostics.

To account for the differences in body size, we tested allometric scaling of clearance and volume parameters with a fixed exponent of 0.75 and 1, respectively, and we tested weight, fat-free mass, or fat mass as body size descriptor (21). After accounting for body size differences, we tested the effect of age, companion PI, NRTI backbone, formulation, and time of dosing (morning vs. evening dose) on the population pharmacokinetic parameters of ritonavir. Covariates were maintained if they reached statistical significance of *P* < 0.05 on forward addition and *P* < 0.01 on backward elimination. We modeled the ritonavir data for each bPI sequentially, starting with the children on darunavir, lopinavir, and then atazanavir, attempting to correct for any systematic differences between the bPIs when including the new arm to the model, as previously suggested (22). The performance of intermediate and final models was assessed both numerically and using goodness of fit plots, and by simulation-based diagnostics such as visual predictive checks (VPC) (23). Parameter precision was evaluated using the sampling importance resampling (SIR) procedure (24). The effect of covariates on the simulated area under the curve from 0 to 24 hours (AUC_0–24 h_) for all study arms was visualized using a forest plot based on 1,000 simulations accounting for parameter uncertainty (25).

## RESULTS

### Study population

Between January 2019 and March 2021, a total of 170 children were enrolled into the lopinavir/ritonavir, atazanavir/ritonavir, and darunavir/ritonavir arms of the CHAPAS-4 pharmacokinetic sub-study. Median age was 10.6 (range 3.16–15.6) years, weight 26.0 (14.2–64.2) kg, and 56% were female. There was a total of 1,254 ritonavir concentrations: 6.9% were below the LLOQ, but only 3.7% were undetectable. One participant’s profile was excluded because of drug concentrations implausibly low throughout the profile (close to LLOQ), and it was suspected that this was due to a lack of adherence or other issues with drug dosing. We excluded all 24 hour pharmacokinetic samples for children on lopinavir/ritonavir because the 12 hour dosing times were insufficiently documented, which made model fitting of these samples challenging. Table 1 summarizes the baseline characteristics for all children on ritonavir.

### Population pharmacokinetic analysis

A two-compartment disposition model (change in objective function value [dOFV] = −110, *P* < 0.001, two extra degrees of freedom compared to one-compartment disposition) with a lag time in absorption followed by sequential zero- and first-order absorption (dOFV = −488, *P* < 0.001, two extra degrees of freedom compared to a model with no delay in absorption) best described ritonavir concentration-time data. First-pass hepatic extraction improved the model fit (dOFV = −10.0, no extra degrees of freedom) but was almost entirely driven by two outlying participants, so it was not retained in the model. Saturable liver elimination did not significantly improve the model fit (dOFV = −0.390, *P* > 0.05), and the estimate of the ritonavir concentration at 50% of the liver’s maximum capacity to metabolize ritonavir (Michaelis constant [Km]) was close to the maximum observed concentration in this study, suggesting no meaningful saturation at the concentrations observed in the study. Fat-free mass-based allometric scaling of clearance and volume parameters was preferred over weight-based scaling (dOFV = −5.0, compared to weight).

We found a significant effect of the companion PIs on ritonavir exposure. Using children on darunavir/ritonavir as the reference group, those on atazanavir/ritonavir had 137% higher bioavailability (dOFV = −71.8; 95% confidence interval (CI) 107%–190%; *P* < 0.001) and those on lopinavir/ritonavir had 23.4% lower bioavailability (dOFV = −14.6; 95% CI 8.20%–34.4%; *P* < 0.001). We observed 20.0% faster clearance (dOFV = −41.1; 95% CI 11.3%–31.3%; *P* < 0.001) in children on atazanavir/ritonavir. Finally, the absorption of ritonavir when taken as an evening dose (lopinavir/ritonavir was dosed twice daily) was 3.61-fold slower (dOFV = −19.9; 95% CI 2.57-fold to 4.56-fold; *P* < 0.001). After accounting for these effects, we found no significant effect of NRTI backbone or age on ritonavir population pharmacokinetics.

**Fig 1:**
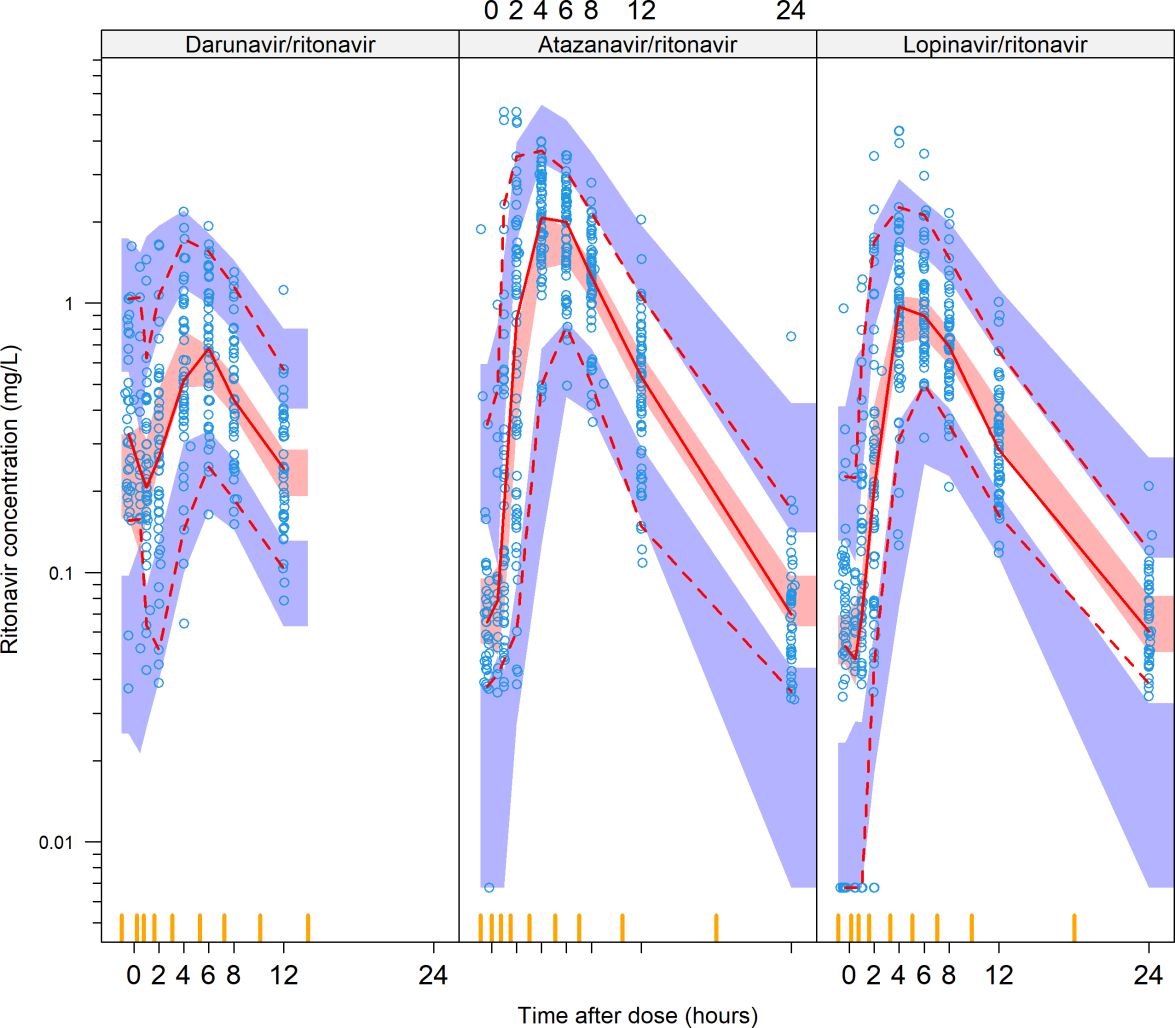
Visual predictive check stratified by boosted protease inhibitor. The solid and dashed lines represent the 5th, 50th, and 95th percentiles of the observed data, while the shaded areas represent the model-predicted 95% confidence intervals for the same percentiles. The circles are the observed concentrations. The outliers in the darunavir arm had taken the previous dose at night while everyone else took it in the morning.

**Fig 2:**
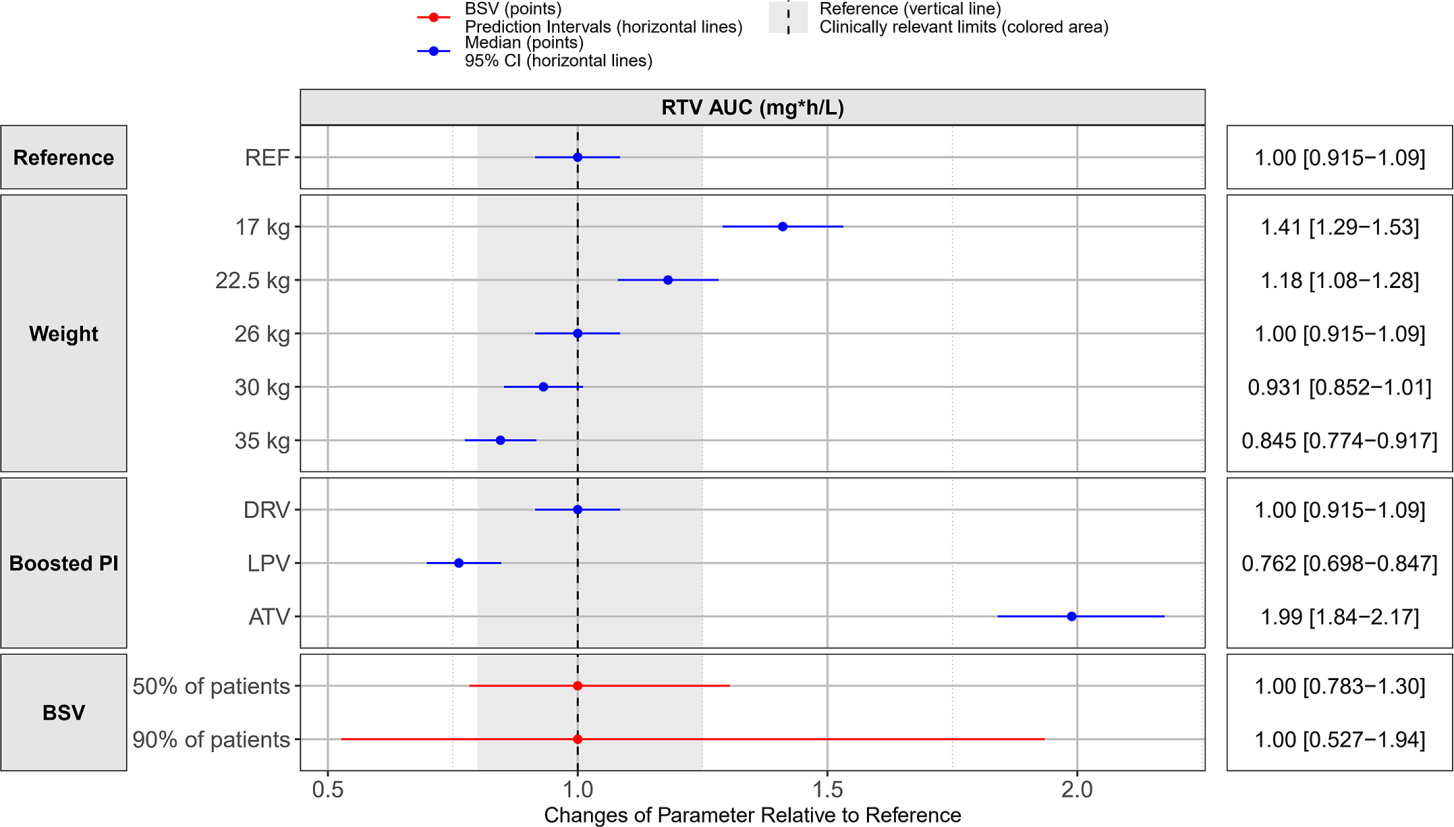
Covariate effects plot showing the relative differences in ritonavir (RTV) area under the curve (AUC_0–24 h_) among different covariates. The reference child has a weight of 26 kg and is on 100 mg ritonavir and 800 mg darunavir. All children in different weight categories are on 100 mg ritonavir. Abbreviations: BSV; between subject variability (50% and 90% indicate the percentage of patients whose AUC_0-24h_ falls within the range defined by the red lines), PI, protease inhibitor; NRTI, nucleoside reverse transcriptase inhibitors; REF, reference; DRV, darunavir; LPV, lopinavir; ATV, atazanavir.

The final population pharmacokinetic parameter estimates, and the model’s fit to the data are shown in Table 3 and Fig. 1, respectively. The effect of the identified covariates on key pharmacokinetic exposure metrics AUC_0–24 h_ and C_max_ is visually presented in Fig. 2. Median (interquartile range) model-based AUC_0–24 h_ and observed C_max_ in our population were 11.3 (7.55–17.2) mg·h/L and 1.37 (0.81–2.12) mg/L, respectively. The model-based AUC_0–24 h_ and observed C_max_ stratified by bPI are shown in Table 4.

**TABLE 3:** Final ritonavir population pharmacokinetic model parameters

Parameter (units)	Typical values (95% confidence interval^a^)
Clearance (L/h)**^b^**	10.5 (9.22–11.9)
Central volume of distribution (L)**^b^**	54.5 (48.9–60.4)
Intercompartmental clearance (L/h)**^b^**	1.19 (0.851–1.58)
Peripheral volume of distribution (L)**^b^**	134 (38.8–330)
Bioavailability	1 FIXED
Lag time (h)	0.981 (0.934–1.06)
Zero-order absorption duration (h)	2.45 (2.05–2.80)
First-order absorption rate constant (1/h)	1.16 (0.893–1.89)
Covariate effects	
Night dose additional lag time (Fold)	3.61 (2.57–4.56)
Lopinavir on relative bioavailability (%)	−23.4 (−8.20 to −34.4)
Atazanavir on relative bioavailability (%)	137 (107–190)
Atazanavir on clearance (%)	+20.7 (+11.3 to +31.3)
Between-subject variability	
Clearance (%)	13.1 (10.2–15.8)
Between-occasion variability	
Bioavailability (%)	40.1 (35.2–46.0)
Lag time (%)	62.0 (54.5–70.2)
Zero-order rate of absorption (%)	52.5 (42.7–63.0)
First-order absorption rate constant (%)	114 (90.0–141)
Extra variability for unobserved doses^c^ (fold change)	2.14 (1.53–2.42)
Residual unexplained variability	
Proportional error (%)	14.7 (13.4–16.1)
Additive error (mg/L)	0.0152 (0.0121–0.0184)

^a^ Based on sampling importance resampling (SIR).

^b^ All clearances and volumes of distribution were allometrically scaled, and the typical values reported here refer to a child weighing 26 kg.

^c^ This parameter was on between-occasion variability in bioavailability.

**TABLE 4:** Model-based area under the curve (AUC_0–24 h_) and observed maximum concentration (C_max_) of ritonavir, stratified by boosted protease inhibitor and weight band

Median (interquartile range)								
Weight-bands	Lopinavir/ritonavir (*N* = 51)		Atazanavir/ritonavir (*N* = 60)		Darunavir/ritonavir (*N* = 59)		All (*N* = 170)	
	AUC_0–24 h_	C_max_	AUC_0–24 h_	C_max_	AUC_0–24 h_	C_max_	AUC_0–24 h_	C_max_
14–19.9 kg	8.57 (5.99–10.1)	0.54 (0.29–0.66)	19.8 (14.7–26.4)^*a*^	2.68 (2.33–3.25)^*a*^	11.8 (7.80–15.3)	1.63 (1.01–2.03)	12.5 (8.70–18.8)	1.55 (0.75–2.52)
20–24.9 kg	10.3 (7.30–12.8)	0.52 (0.42–0.97)	14.7 (11.0–20.5)^*b*^	2.30 (1.89–3.34)^*b*^	9.68 (7.80–12.8)	1.27 (1.10–1.52)	11.4 (8.12–15.5)	1.32 (0.94–2.20)
25–34.9 kg	11.3 (7.29–14.3)	1.04 (0.53–1.36)	20.3 (18.7–24.7)	2.76 (2.29–3.08)	7.86 (6.95–10.6)	1.14 (0.77–1.64)	12.7 (7.78–19.4)	1.64 (1.00–2.62)
≥35 kg	9.83 (7.80–15.3)	0.80 (0.47–1.42)	14.2 (10.6–17.5)	1.76 (1.50–2.28)	6.95 (4.95–7.70)	0.81 (0.53–1.04)	9.52 (7.02–15.2)	1.07 (0.64–1.56)
All	9.83 (6.59–13.2)	0.66 (0.43–1.15)	17.6 (11.7–22.4)	2.38 (1.91–3.03)	8.31 (6.90–11.4)	1.12 (0.87–1.62)	11.3 (7.55–17.2)	1.37 (0.81–2.12)

^*a*^ 3 out of 12 children in this weight band got 100 mg ritonavir instead of 75 mg.

^*b*^ 1 out of 16 children in this weight band got 100 mg ritonavir instead of 75 mg.

## DISCUSSION

In this analysis, we characterized the population pharmacokinetics of ritonavir in children using bPIs as second-line ART within the CHAPAS-4 study. Our final model parameters are comparable with previously reported pediatric ritonavir population pharmacokinetic models (11, 26). We then explored the effects of different covariates on the exposure of ritonavir and found differences in bioavailability and clearance among the children on different bPIs. Compared to children on darunavir/ritonavir, those on atazanavir/ritonavir more than doubled bioavailability and slightly reduced clearance, while those on lopinavir/ritonavir had reduced bioavailability. Importantly, we did not identify any significant effect of TAF/FTC or other NRTIs on ritonavir exposure.

The impact of these effects causes differences in exposures of ritonavir (Fig. 2). Choosing as reference a child of 26 kg receiving 100 mg of ritonavir in combination with darunavir, the co-administration with lopinavir lowers ritonavir AUC_0–24 h_ by 34.8%, while atazanavir increases ritonavir AUC_0–24 h_ by 99.0%. Because of the effect of body size on clearance, AUC_0–24 h_ increases with decreasing weight when the dose remains constant, so metabolically mature children receiving the same dose as adults (100 mg) experience higher exposures.

The precise mechanism causing higher ritonavir bioavailability when given with atazanavir is not fully clear, but previous studies have shown that atazanavir may increase the exposure of other protease inhibitors (including ritonavir) through its inhibition of CYP3A4 (27–29). Additionally, we hypothesize that another mechanism may be through differences in affinity to CYP3A4 and P-glycoprotein transporters among the bPIs (13, 14). This may lead to ritonavir having higher bioavailability when given with atazanavir, if atazanavir has relatively higher affinity to P-glycoprotein transporters compared to other bPIs. Simultaneously, we cannot rule out the possibility that our reference arm (darunavir) and/or lopinavir might be reducing the bioavailability of ritonavir. A key challenge in investigating this phenomenon is the absence of data for ritonavir administered alone. However, a cross-over pharmacokinetic study reported that darunavir lowered the plasma AUC of ritonavir by 14% (30).

We also considered the possibility that this finding of higher ritonavir bioavailability in children on atazanavir/ritonavir could be a result of differences in the ritonavir formulations used across the bPIs. Children on atazanavir/ritonavir received the co-formulated 300/100 mg atazanavir/ritonavir if they weighed ≥25 kg. If they weighed <25 kg, they were given a single tablet of 100 mg atazanavir with 75 mg ritonavir (three 25 mg ritonavir tablets) or a single 100 mg ritonavir tablet if the 25 mg ritonavir tablets were not available. When the atazanavir effect on bioavailability was split by ritonavir formulation instead, we found that all children on atazanavir showed higher bioavailability, regardless of formulation, including those who happened to receive the same 100 mg ritonavir formulation (four children) used in the darunavir arm. This observation suggests that the observed differences in bioavailability are not attributable to formulation effects.

The main CHAPAS-4 study reported significantly higher events of grade 3–4 hyperbilirubinemia in the atazanavir/ritonavir arm compared with other PI arms (16). Atazanavir is known to cause hyperbilirubinemia through competitive inhibition of UGT1A1, and concomitant ritonavir exacerbates this side effect. This effect might be further exacerbated by the higher ritonavir exposure due to atazanavir (31). It might be worth exploring lower doses of ritonavir to boost atazanavir to mitigate this side effect while maintaining sufficient exposure to boost atazanavir (32).

In the children atazanavir/ritonavir, we also observed 20% higher ritonavir clearance, an effect that might seem contradictory to the finding of higher bioavailability mentioned above. However, previous reports have shown that atazanavir may increase the activity of hepatic CYP3A4 to a higher degree than lopinavir and other protease inhibitors, and this could also be the mechanism at play (12).

In contrast to the higher ritonavir bioavailability in the children on atazanavir, we observed a 23.4% lower ritonavir bioavailability in children on lopinavir/ritonavir. Previous studies have indicated that lopinavir reduces ritonavir exposure, and this effect is amplified when higher doses of lopinavir are administered alongside a fixed dose of ritonavir (29, 33). Additionally, we cannot exclude that lower bioavailability in children on lopinavir/ritonavir might be due to the co-formulated lopinavir/ritonavir formulation.

Finally, we observed C_0_ (drug concentrations just before the observed dose) higher than C_12_ (drug concentrations 12 hours after the observed dose). We accounted for this in the model by including an effect of time of dose (day or night) on ritonavir’s speed of absorption. Children on lopinavir/ritonavir took their bPI dose twice a day, and we observed that the doses taken at night took 3.6 times longer to be absorbed than the doses taken during the day. This effect has been previously reported by HSU et al., who found that the evening ritonavir T_max_ was 1.7 hours later than when ritonavir was given during the day (4). The possible explanation for this delay in night absorption could be a bigger meal given with the doses at night, or lower gastrointestinal blood flow during sleep, which could delay absorption (34, 35). The slow absorption at night is not expected to affect ritonavir’s ability to boost lopinavir once at steady state.

Our study had some strengths and weaknesses. To the best of our knowledge, this is the first study to directly compare ritonavir exposures when co-administered with different protease inhibitors within a single study. This allowed for consistent procedures across the various boosted PI arms, which strengthened the comparability of the results. For instance, the blood sampling times, drug assay, study sites, and food intake were the same among the bPI arms. This uniformity made the pharmacokinetic analysis of the ritonavir data more robust by reducing unexplained variability, and it allowed us to identify differences between the bPIs. Our main limitation was that we did not have data on ritonavir alone to serve as a control group. As a result, we used one of the bPI arms (darunavir/ritonavir) as a reference group and reported the difference relative to the reference. Additionally, changes in ritonavir dose coincided with variations in the companion PI, formulation, and patient weight, limiting our ability to assess dose/exposure nonlinearity and fully separate the formulation effect from that of the PI. Although we evaluated the formulation effect, the PI’s impact was more pronounced. Finally, we only had data on ritonavir at the dose levels used for boosting other PIs, and as a result, we could not characterize the saturation of clearance reported at higher doses. This means that our model may not be suitable to simulate doses of ritonavir above 100 mg.

In conclusion, we characterized ritonavir pharmacokinetics in children on different bPIs and found differences in ritonavir bioavailability and clearance among the bPIs, which is in line with previous adult studies (29). Future studies could explore optimizing and customizing doses of ritonavir used to boost the respective PIs. There was no significant effect of TAF/FTC or other NRTIs on ritonavir exposure. These data strengthen the evidence base and enhance our understanding of the population pharmacokinetics of ritonavir administered to boost lopinavir, atazanavir, or darunavir in children with HIV in Africa.

## SUPPLEMENTAL MATERIAL

Data S1 aac.00771-25-s0001.docx Model code and clinical trial team composition.

ASM does not own the copyrights to Supplemental Material that may be linked to, or accessed through, an article. The authors have granted ASM a non-exclusive, world-wide license to publish the Supplemental Material files. Please contact the corresponding author directly for reuse.
