# Supplementary material for: Population pharmacokinetics of ritonavir as a booster of lopinavir, atazanavir, or darunavir in African children with HIV
Source: Antimicrob Agents Chemother. 2025 Sep 26;69(11):e00771-25. doi: 10.1128/aac.00771-25 (PMC12587601; doi:10.1128/aac.00771-25)
Supplement: Data S1 — Model code and clinical trial team composition. [file aac.00771-25-s0001.docx]

**Population pharmacokinetics of ritonavir as a booster of lopinavir, atazanavir, or darunavir in African children with HIV.**

Lufina Tsirizani^1,2^, Hylke Waalewijn^1^, Alexander Szubert^3^, Veronica Mulenga^4^, Chishala Chabala^1,4,5^, Mutsa Bwakura-Dangarembizi^6,7^, Moses Chitsamatanga^6^, Diana A. Rutebarika^8^, Victor Musiime^8,9^, Mariam Kasozi^10^, Abbas Lugemwa^10^, Helen M. McIlleron^1,11^, David M. Burger^12^, Diana M. Gibb^3^, Angela Colbers^12^, Paolo Denti^1^, Roeland E. Wasmann^1#^, the CHAPAS-4 trial team.

**AFFILIATIONS:**

1. Division of Clinical Pharmacology, Department of Medicine, University of Cape Town, Cape Town, South Africa
2. Training and Research Unit of Excellence, Kamuzu University of Health Sciences, Malawi
3. Medical Research Council Clinical Trials Unit at University College London, London, United Kingdom
4. University of Zambia, School of Medicine, Department of Paediatrics and Child Health, Lusaka, Zambia
5. University Teaching Hospital, Lusaka, Zambia
6. University of Zimbabwe Clinical Research Centre, Harare, Zimbabwe
7. University of Zimbabwe Faculty of Medicine and Health Sciences, Department of Child, Adolescent and Women’s Health
8. Department of paediatrics, joint Clinical Research Centre, Kampala, Uganda
9. Department of paediatrics and Child Health, Makerere University, College of Health Sciences, School of Medicine, Kampala, Uganda
10. Department of HIV research, joint Clinical Research Centre, Mbarara, Uganda
11. Wellcome Centre for Infectious Diseases Research in Africa (CIDRI-Africa), Institute of Infectious Disease and Molecular Medicine, University of Cape Town, Cape Town, South Africa
12. Department of Pharmacy, Radboudumc Research Institute for Medical Innovation (RIMI), Radboud University Medical Center, Nijmegen, the Netherlands

**NONMEM code for ritonavir population pharmacokinetic model**

$SIZES PD=-1000 LVR=-150 LTH=-200 MAXFCN=10000000 LNP4=-150000

$PROBLEM PAED_RTV

$INPUT ID NRTI PI_BCK_BONE ART_REG DRUG

DAT2=DROP TIME EVID OCC DV MDV AMT BLQ

CENS RATE AGE SEX HT WT

WT_BAND DRUG_CODE DOSE_PI_DTG TAF_DOSE RTV_MG_L_ORIG PROBLEM SERUM_ALT SERUM_AST SERUM_TOTAL_BILIRUBIN

SERUM_CRETININE SERUM_UREA CREATININE_CLEARANCE SERUM_PHOSPHATE

ALBUMIN URINE_CREATININE PROTEIN URINE_PHOSPHATE RTV_DOSE

TOTAL_RTV_DOSE FORMULATION FLAG_DRV FLAG_ATV VPC_TIME FFM

$DATA RTV_data.csv IGNORE=@

$ABBREVIATED DERIV2=NO

$SUBROUTINE ADVAN4 TRANS1 ; 2 compartments

;------------------------------------------------------------------------------

$PK

A_0(1) = 0.0000001

A_0(2) = 0.0000001

A_0(3) = 0.0000001

;RE-ASSIGIN THE ETA VARIABLES

BOVCL = 0

BOVBIO = 0

BOVKA = 0

BOVLAG = 0

BOVD1 = 0

EBOV = THETA(13) ; Extra BOV for unobserved doses

; ---------- BOV

IF(OCC.EQ.1)BOVCL = ETA(7)

IF(OCC.EQ.2)BOVCL = ETA(8)

IF(OCC.EQ.1)BOVBIO = ETA(9)*EBOV

IF(OCC.EQ.2)BOVBIO = ETA(10)

IF(OCC.EQ.1)BOVKA = ETA(11)

IF(OCC.EQ.2)BOVKA = ETA(12)

IF(OCC.EQ.1)BOVLAG = ETA(13)

IF(OCC.EQ.2)BOVLAG = ETA(14)

IF(OCC.EQ.1)BOVD1 = ETA(15)

IF(OCC.EQ.2)BOVD1 = ETA(16)

; ------- BSV

BSVCL = ETA(1)

BSVV = ETA(2)

BSVKA = ETA(3)

BSVBIO = ETA(4)

BSVV3 = ETA(5)

BSVQ = ETA(6)

; ------- Typical values of covariates

TVFFM = 21.0 ;MEDIAN

;--------- Allometric scaling and covariates

ALLMCL_FFM = (FFM/TVFFM)**0.75

ALLMV_FFM = (FFM/TVFFM)

;---LPV night ON MTT------

LPV_NIGHT_LAG = 1

IF(PI_BCK_BONE.EQ.3.AND.OCC.EQ.1)LPV_NIGHT_LAG = THETA(10)

;---LPV on BIO------

LPV_BIO = 1

IF(PI_BCK_BONE.EQ.3)LPV_BIO = THETA(11)

;---ATV on BIO------

ATV_BIO = 1

IF(PI_BCK_BONE.EQ.2)ATV_BIO = THETA(12)

;---ATV on CL------

ATV_CL = 1

IF(PI_BCK_BONE.EQ.2)ATV_CL = THETA(15)

;---------Typical values-----------

TVCL = THETA(1)*ALLMCL_FFM*ATV_CL

TVV = THETA(2)*ALLMV_FFM

TVKA = THETA(3)

TVBIO = THETA(4)*LPV_BIO*ATV_BIO

TVV3 = THETA(7)*ALLMV_FFM

TVQ = THETA(8)*ALLMCL_FFM

TVLAG = THETA(9)*LPV_NIGHT_LAG

TVD1 = THETA(14)

;-----------------------------------------

;-----------Define parameters------

CL = TVCL*EXP(BSVCL+BOVCL) ; CLEARANCE

V = TVV*EXP(BSVV) ; CENTRAL VOL.

KA = TVKA*EXP(BSVKA+BOVKA) ; ABS. RATE CONSTANT

BIO = TVBIO*EXP(BSVBIO+BOVBIO) ; BIOAVAILABILITY

V3 = TVV3*EXP(BSVV3) ; PERIPH VOL

Q = TVQ*EXP(BSVQ) ; INTER COMPT CL

LAG = TVLAG*EXP(BOVLAG) ; LAG

D1 = TVD1*EXP(BOVD1)

;-------------------------------------------------------------------------------------------------------------------------------------

ALAG1=LAG

F1 = BIO

S2 = V ;CENTRAL COMPARTMENT SCALAR (based on numbering in $MODEL)

K = CL/V ;(rate constant of elimination)

K23 = Q/V ; (rate constant from central to peripheral 1)

K32 = Q/V3 ;(rate constant from peripheral 1 to central)

;-------------------------------------------------------------------------------------------------------------------------------------

$ERROR

IPRED=A(2)/V

; DEFINE LLOQ VALUE

LLOQ=0.045

CENS_THR = 0.3*LLOQ ; 30% of LLOQ

PROP = IPRED*THETA(5)

ADD = THETA(6)+(LLOQ*0.2)

IF (ICALL/=4.AND.CENS==1) THEN

ADD = ADD +(CENS_THR*0.5)

ENDIF

NO_FIT = 0

IF (ICALL/=4.AND.CENS==2) THEN

PROP = 0

ADD = 10000000000

NO_FIT = 1

ENDIF

W = SQRT(ADD**2+PROP**2)

; Protective code

IF (W.LE.0.000001) W=0.000001

IRES=DV-IPRED

IWRES=IRES/W

Y = IPRED + W*ERR(1)

IF (ICALL==4.AND.Y<=CENS_THR) Y = CENS_THR/2

;--------------------------RETRIEVE AMOUNT IN EACH COMPARTMENT-------------------

AA1 = A(1) ;ABS CMT

AA2 = A(2) ;CENTRAL CMT

AA3 = A(3) ;PERI

;------------------------------------------------------------------------------------------------------------------

$THETA (0,10.3447,20) ; 1 CL [L/h]

$THETA (0,53.669,500) ; 2 V [L]

$THETA (0,1.13641,10) ; 3 KA [1/h]

$THETA 1 FIX ; 4 BIO

$THETA (0,0.152184,1) ; 5 PROP []

$THETA (0,0.00647417,1) ; 6 ADD [mg/L]

$THETA (0,129.191,800) ; 7 V3 [L]

$THETA (0,1.13625,10) ; 8 Q [L/h]

$THETA (0,0.995255,20) ; 9 LAG [h]

$THETA (0,2.93317,5) ; 10 LPV_NIGHT_LAG

$THETA (0,0.779626,5) ; 11 LPV_BIO

$THETA (0,2.32224,10) ; 12 ATV_BIO

$THETA (0,2.10111,5) ; 13 EBOV

$THETA (0,2.26624,30) ; 14 D1

$THETA (0,1.20875,5) ; 15 ATV_CL

;-------------------------------------------------------------------------------------------------------------------------------------

$OMEGA BLOCK(1)

0.0154914 ; 1 BSVCL

$OMEGA BLOCK(1) FIX

0 ; 2 BSV V

$OMEGA BLOCK(1) FIX

0 ; 3 BSV KA

$OMEGA BLOCK(1) FIX

0 ; 4 BSV BIO

$OMEGA BLOCK(1) FIX

0 ; 5 BSVV3

$OMEGA BLOCK(1) FIX

0 ; 6 BSVQ

;-------------------------------------------------------------------------------------------------------------------------------------

$OMEGA BLOCK(1) FIX

0 ; 7 BOVCL

$OMEGA BLOCK(1) SAME

;-------------------------------------------------------------------------------------------------------------------------------------

$OMEGA BLOCK(1)

0.159089 ; 9 BOVBIO

$OMEGA BLOCK(1) SAME

;-------------------------------------------------------------------------------------------------------------------------------------

$OMEGA BLOCK(1)

1.25373 ; 11 BOVKA

$OMEGA BLOCK(1) SAME

;-------------------------------------------------------------------------------------------------------------------------------------

$OMEGA BLOCK(1)

0.372121 ; 13 BOVLAG

$OMEGA BLOCK(1) SAME

;-------------------------------------------------------------------------------------------------------------------------------------

$OMEGA BLOCK(1)

0.289698 ; 15 BOVD1

$OMEGA BLOCK(1) SAME

;-------------------------------------------------------------------------------------------------------------------------------------

$SIGMA 1 FIX

;-------------------------------------------------------------------------------------------------------------------------------------

$ESTIMATION MSFO=run001.msf MAXEVAL=9999 PRINT=1 METHOD=1 INTER

NOABORT NSIG=3 SIGL=6 NONINFETA=1 ETASTYPE=1 SORT

$COVARIANCE PRINT=E MATRIX=R

$TABLE ID OCC TIME VPC_TIME TAD Y DV MDV EVID AA1 AA2 AA3 PRED

RES WRES IPRED IRES IWRES CWRESI CWRES OBJI CL V KA BIO V3

Q LAG BSVCL BSVQ BOVCL BOVKA BOVBIO BOVLAG BOVD1 AGE WT

FFM FAT HT SEX NRTI WT_BAND DRUG TAF_DOSE SERUM_ALT

SERUM_AST SERUM_TOTAL_BILIRUBIN SERUM_CRETININE SERUM_UREA

CREATININE_CLEARANCE ALBUMIN URINE_CREATININE PROTEIN

URINE_PHOSPHATE RTV_DOSE PI_BCK_BONE

TOTAL_RTV_DOSE FORMULATION FLAG_DRV FLAG_ATV DOSE_PI_DTG

ART_REG FILE=mytab001.csv NOPRINT NOAPPEND ONEHEADER

FORMAT=,

**CHAPAS-4 Trial team composition**

Clinical Trials Unit:

MRC CTU at UCL

Di Gibb, Sarah Walker, Anna Turkova, Clare Shakeshaft, Moira Spyer, Margaret Thomason, Anna Griffiths, Lara Monkiewicz, Sue Massingham, Alex Szubert, Alasdair Bamford, Katja Doerholt, Amanda Bigault, Nimisha Dudakia, Annabelle South, Nadine Van Looy, Carly Au, Hannah Sweeney

Trial Sites:

Joint Clinical Research Centre Lubowa, Uganda: Cissy M. Kityo, Victor Musiime, Eva Natukunda, Esether Nambi, Diana Rutebarika Antonia, Rashida Nazzinda, Imelda Namyalo, Joan Nangiya, Lilian Nabeeta, Aidah Nakalyango, Lilian Kobusingye, Caroline Otike, Winnie Namala, Phionah Ampaire, Ayesiga Edgar, Claire Nasaazi, Milly Ndigendawani, Paul Ociti, Priscilla Kyobutungi, Ritah Mbabazi, Phyllis Mwesigwa Rubondo, Juliet Ankunda, Mariam Naabalamba, Mary Nannungi, Alex Musiime, Faith Mbasani, Babu Enoch Louis, Josephine Namusanje, Denis Odoch, Edward Bagirigomwa, Eddie Rubanga, Disan Mulima, Paul Oronon, Eram David Williams, David Baliruno, Josephine Kobusingye, Agnes Uyungrwoth, Barbara Mukanza, Jimmy Okello, Emily Ninsiima, Lutaro Ezra, Christine Nambi, Nansaigi Mangadalen, Musumba Sharif, Nobert B. Serunjogi, Otim Thomas

Joint Clinical Research Centre Mbarara, Uganda: Abbas Lugemwa, Shafic Makumbi, Sharif Musumba, Edward Mawejje, Ibrahim Yawe, Linda Jovia Kyomuhendo, Mariam Kasozi, Rogers Ankunda, Samson kariisa, Christine Inyakuwa, Emily Ninsiima, Lorna Atwine, Beatrice Tumusiime, John Ahuura, Deogracious Tukwasibwe, Violet Nagasha, Judith Kukundakwe, Mariam Zahara Nakisekka, Ritah Winnie Nambejja, Mercy Tukamushaba, Rubinga Baker, Edridah Keminyeto, Barbara Ainebyoona, Sula Myalo, Juliet Acen, Nicholas Jinta Wangwe, Ian Natuhurira, Gershom Kananura Natukunatsa

University Teaching Hospital, Zambia: Veronica Mulenga, Chishala Chabala, Joyce Chipili Lungu, Monica Kapasa, Khonzya Zyambo, Kevin Zimba, Dorothy Zangata, Ellen shingalili, Naomi Mumba, Nayunda kaonga, Mukumbi Kabesha, Oliver Mwenechanya, Terrence Chipoya, Friday Manakalanga, Stephen Malama, Daniel Chola

Arthur Davison Children’s Hospital, Zambia: Bwendo Nduna, Mwate Mwamabazi, Kabwe Banda, Beatrice Kabamba, Muleya Inambao, Pauline Mahy Mukandila, Mwizukanji Nachamba, Stella Himabala, Shadrick Ngosa, Davies Sondashi, Collins Banda, Mark Munyangabe, Grace Mbewe Ngoma, Sarah Chimfwembe, Mercy Lukonde Malasha, Mumba Kajimalwendo, Henry Musukwa, Shadrick Mumba

University of Zimbabwe Clinical Research Centre, Zimbabwe: James Hakim, Mutsa Bwakura-Dangarembizi, Kusum Nathoo, Taneal Kamuzungu, Ennie Chidziva, Joyline Bhiri, Joshua Choga, Hilda Angela Mujuru, Godfrey Musoro, Vivian Mumbiro, Moses Chitsamatanga, Constantine Mutata, Rudo Zimunhu, Shepherd Mudzingwa, Secrecy Gondo, Columbus Moyo, Ruth Nhema, Kathryn Boyd, Farai Matimba, Vinie Kouamou, Richard Matarise, Zorodzai Tangwena, Taona Mudzviti, Allen Matubu, Alfred Kateta, Victor Chinembiri, Dorinda Mukura, Joy Chimanzi, Dorothy Murungu, Wendy Mapfumo, Pia Ngwaru, Lynette Chivere, Prosper Dube, Trust Mukanganiki, Sibusisiwe Weza, Tsitsi Gwenzi, Shirley Mutsai, Misheck Phiri, Makhosonke Ndlovu, Tapiwa Gwaze, Stuart Chitongo, Winisayi Njaravani, Sandra Musarurwa, Cleopatra Langa, Sue Tafeni, Wilbert Ishemunyoro, Nathalie Mudzimirema

Mpilo Central Hospital, Zimbabwe: Wedu Ndebele, Mary Nyathi, Grace Siziba, Getrude Tawodzera, Tracey Makuchete, Takudzwa Chidarura, Shingaidzo Murangandi, Lawrence Mafaro, Owen Chivima, Sifiso Dumani, Beaullar Mampondo, Constance Maphosa, Debra Mwale, Rangarirai Dhlamini, Thabani Sibanda, Nobukhosi Madubeko, Silibaziso Nyathi, Zibusiso Matiwaza

Local External Site Monitors:

Uganda: Sylvia Nabukenya, Harriet Tibakabikoba, Sarah Nakalanzi, Cynthia Williams

Zimbabwe: Precious Chandiwana, Winnie Gozhora, Benedictor Dube

Zambia: Sylvia Mulambo, Hope Mwanyungwi

Sub-studies:

PK sub-studies – Radboud University Medical Centre: David Burger, Angela Colbers, Hylke Waalewijn, Lisanne Bevers, Shaghayegh Mohsenian-Naghani

PK sub-studies – University of Cape Town: Helen McIlleron, Jennifer Norman, Lubbe Wiesner, Roeland Wasmann, Paolo Denti, Lufina Tsirizani Galileya

Toxicity sub-study: Eva Natukunda, Victor Musiime, Phillipa Musoke

Health Economics sub-study – University of York: Paul Revill, Simon Walker

Trial Committees:

Independent Trial Steering Committee Members: Adeodata Kekitiinwa, Angela Mushavi, Febby Banda Kawamya, Denis Tindyebwa, Hermione Lyall, Ian Weller

Independent Data Monitoring Committee Members: Tim Peto, Philippa Musoke, Margaret Siwale, Rose Kambarami

Funders:

EDCTP: Johanna Roth, Pauline Beattie
